# Supplementary material for: Content-rich biological network constructed by mining PubMed abstracts
Source: BMC Bioinformatics. 2004 Oct 8;5:147. doi: 10.1186/1471-2105-5-147 (PMC528731; doi:10.1186/1471-2105-5-147)
Supplement: Additional File 2 — The original results of the above study (non-essential files are deleted to keep the file size under the limit set by BMC bioinformatics). [file 1471-2105-5-147-S2.bz2 › chilibotAdditionalFile2/dip05/378ID9927421E9791/html/NGFR_NTRK2.html]

 


 **NGFR** and **NTRK2** 
  
Found 102 abstracts in PubMed, retrieved 05.  
 

 What does Google say? 
 PDF only 
| .edu only 

---

**Interactive relationship** (e.g. stimulation, inhibition, etc)

**Neutral relationship**- Although neurotrophins did not regulate p75NTR  [ **NGFR** ]  processing, the alpha and gamma secretase mediated cleavage of p75 is modulated by receptor tyrosine kinases Trks TrkA and TrkB  [ **NTRK2** ]  but not TrkC.  Ref: 12843241 J Neurosci, 2003

**Non-interactive relationship** (e.g. studied together, co-existance, homology, etc.)

- Activation of the TrkB  [ **NTRK2** ]  and the p75NTR  [ **NGFR** ]  by the addition of BDNF facilitates neuronal commitment and marked neurite genesis.  Ref: 12589813 Biochem Biophys Res Commun, 2003
- We have used immunofluorescence techniques with a panel of antibodies against known neurotrophin receptors trk A, trk B  [ **NTRK2** ] , trk C, p75NTR  [ **NGFR** ]  to map the locations of these receptors in the developing neuromuscular system of mice from our neurotrophin 3 NT 3 knockout colony.  Ref: 12141445 Int J Dev Biol, 2002
